# Activity and Life After Survival of a Cardiac Arrest (ALASCA) and the effectiveness of an early intervention service: design of a randomised controlled trial

**DOI:** 10.1186/1471-2261-7-26

**Published:** 2007-08-27

**Authors:** Véronique RMP Moulaert, Jeanine A Verbunt, Caroline M van Heugten, Wilbert GM Bakx, Anton PM Gorgels, Sebastiaan CAM Bekkers, Marc CFTM de Krom, Derick T Wade

**Affiliations:** 1 Rehabilitation Foundation Limburg, Hoensbroek, The Netherlands; 2 Department of General Practice, Maastricht University, Maastricht, The Netherlands; 3 Department Brain and Behavior, Maastricht University, Maastricht, The Netherlands; 4 Utrecht Centre of Excellence for Rehabilitation Medicine de Hoogstraat, Utrecht, The Netherlands; 5 Department of Cardiology, University Hospital Maastricht, Maastricht, The Netherlands; 6 Department of Neurology, University Hospital Maastricht, Maastricht, The Netherlands; 7 Oxford Centre of Enablement, Oxford, UK; 8 Care And Public Health Research Institute, Maastricht University, Maastricht, The Netherlands

## Abstract

**Background:**

Cardiac arrest survivors may experience hypoxic brain injury that results in cognitive impairments which frequently remain unrecognised. This may lead to limitations in daily activities and participation in society, a decreased quality of life for the patient, and a high strain for the caregiver. Publications about interventions directed at improving quality of life after survival of a cardiac arrest are scarce. Therefore, evidence about effective rehabilitation programmes for cardiac arrest survivors is urgently needed. This paper presents the design of the ALASCA (Activity and Life After Survival of a Cardiac Arrest) trial, a randomised, controlled clinical trial to evaluate the effects of a new early intervention service for survivors of a cardiac arrest and their caregivers.

**Methods/design:**

The study population comprises all people who survive two weeks after a cardiac arrest and are admitted to one of the participating hospitals in the Southern part of the Netherlands. In a two-group randomised, controlled clinical trial, half of the participants will receive an early intervention service.

The early intervention service consists of several consultations with a specialised nurse for the patient and their caregiver during the first three months after the cardiac arrest. The intervention is directed at screening for cognitive problems, provision of informational, emotional and practical support, and stimulating self-management. If necessary, referral to specialised care can take place. Persons in the control group will receive the care as usual.

The primary outcome measures are the extent of participation in society and quality of life of the patient one year after a cardiac arrest. Secondary outcome measures are the level of cognitive, emotional and cardiovascular impairment and daily functioning of the patient, as well as the strain for and quality of life of the caregiver. Participants and their caregivers will be followed for twelve months after the cardiac arrest.

A process evaluation will be performed to gain insight into factors that might have contributed to the effectiveness of the intervention and to gather information about the feasibility of the programme. Furthermore, an economic evaluation will be carried out to determine the cost-effectiveness and cost-utility of the intervention.

**Discussion:**

The results of this study will provide evidence on the effectiveness of this early intervention service, as well as the cost-effectiveness and its feasibility.

**Trial registration:**

Current Controlled Trials [ISRCTN74835019]

## Background

The incidence of cardiac arrest is 1 – 2 per 1000 inhabitants a year but differs according to country and region [1]. In the Netherlands, the incidence of cardiac arrests lies between 0.6 – 0.9 per 1000 inhabitants per year, resulting in approximately 16,000 cases of cardiac arrests each year [2,3]. Resuscitation is attempted in 30 – 50% of the cases [4]. The survival rate at discharge from the hospital after attempted resuscitations is only 9 to 16% for out-of-hospital resuscitations [2,5-7] and between 14 and 37% for in-hospital resuscitations [8-11]. Faster access to external automated defibrillators (AEDs) outside the hospital will probably raise survival rates [4,12]. This will lead to an increased number of cardiac arrest survivors and, thus, to more patients having to live with the consequences of surviving a cardiac arrest.

A cardiac arrest may lead to irreversible brain damage, called posthypoxic encephalopathy or hypoxic brain injury. Hypoxic brain injury often leads to cognitive or emotional impairments, with memory disorders and depressive symptoms being the most common complaints [13-15]. As a result of this, the performance of daily activities can be limited even six to twelve months later, with almost a quarter of the people needing some form of assistance in daily life [13,16-19]. Furthermore, participation in society often decreases after a cardiac arrest. For example, of the people who were working prior to a cardiac arrest only 20% were able to resume their job [20,21]. This all seems to result in a reduced quality of life for both the patient and their caregiver and leads to a high strain on the carer [18,20-22].

Unfortunately, although the number of problems is substantial, a large percentage of these problems, especially the cognitive impairments, are not recognised or treated.

Currently, there exist no specific follow-up programmes or nursing interventions for cardiac arrest survivors in the Netherlands. In the international literature, only a few programmes for cardiac arrest survivors were found. In a study by Cowan et al., psychosocial nursing therapy reduced two-year mortality in a group of cardiac arrest survivors [23]. This intervention consisted of physiological relaxation (biofeedback), cognitive behavioral therapy aimed at self-management and health education. Dougherthy et al. showed that an intervention, based on the principles of social cognitive theory and the concerns of patients and their partners, reduced physical symptoms and anxiety and enhanced knowledge in persons who had received an internal cardioverter defibrillator (ICD) implantation [24-26]. However, this intervention had no effect on health care use [25,26].

The diffuse brain injury and cognitive loss after a cardiac arrest seems in many ways similar to that seen after traumatic brain injury. In a study on traumatic brain injury patients, a routine follow up service resulted in a reduction in social morbidity and severity of post concussion symptoms six months later [27]. Given that cognitive impairment is a major problem in both hypoxic brain injury and moderate head injury, a comparable type of treatment could prove beneficial for patients after a cardiac arrest.

### Aims and hypotheses

The primary goal of the present study is to evaluate the effectiveness of a new, early intervention service for survivors of a cardiac arrest and their caregivers. Secondly, a process evaluation will be performed to gain insight into factors that might have contributed to the effectiveness of the intervention and to gather information about the feasibility of the programme. Finally, an economic evaluation will determine the cost-effectiveness and cost-utility of the intervention.

We anticipate that the prospective follow-up and support provided by a specialised nurse will reduce the negative consequences of a cardiac arrest and will result in a higher level of participation in society and quality of life of the cardiac arrest survivor. Furthermore, we expect that this intervention will be cost-effective.

## Methods/Design

### Study design

**Figure 1:**
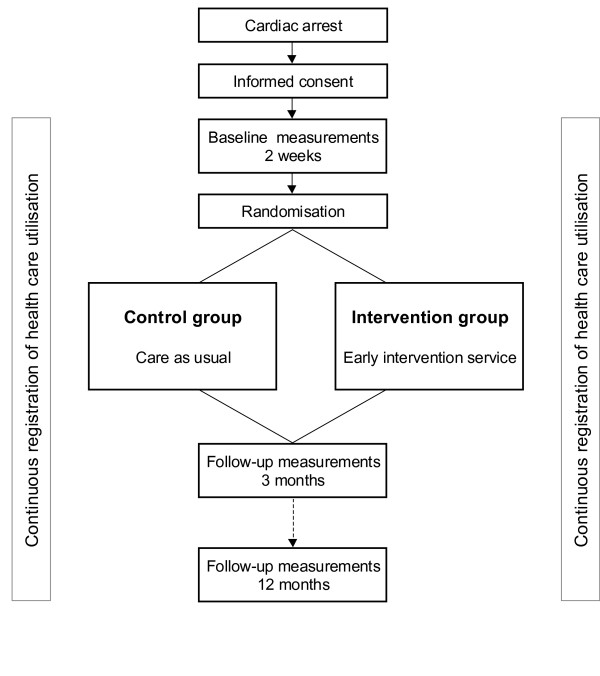
Study design.

The study presented in this paper is a randomised, controlled clinical trial in which the effect of an early intervention service will be investigated. Participants will be followed over one year. During this year, three measurements will take place, namely two weeks, three months and twelve months after the cardiac arrest (see flow diagram, Figure 1). The Medical Ethics Committee of University Hospital Maastricht/Maastricht University approved the study protocol. The study is registered in a public trial registry [ISRCTN74835019]. This study is part of a larger research project, which also comprises a prospective and prognostic cohort study.

### Setting

Participants will be recruited from the coronary care units and intensive care units of four hospitals in the Southern part of the Netherlands, starting in April 2007. One of the participating hospitals is a university hospital.

### Study population

The population of this study consists of cardiac arrest survivors admitted to one of the participating hospitals, including both survivors of out-of-hospital and in-hospital cardiac arrests (for in- and exclusion criteria, see Table 1) and their caregivers. In this study caregiver is defined as partner, spouse, or significant other that is most closely related to the patient.

**Table 1:** Inclusion and exclusion criteria

**Inclusion criteria**
- All people who survive two weeks after a cardiac arrest
- Admitted in or to one of the participating hospitals
- Living within 50 km of one of the participating hospitals
- 18 years or older
- Sufficient knowledge of Dutch language
**Exclusion criteria**
- Life expectancy lower than 3 months (as assessed by the treating physician)
- Living in residential or institutional care prior to the cardiac arrest

The sample size calculation (two groups and one-sided testing) results from research on traumatic brain injured patients with regard to their participation in society assessed using the Community Integration Questionnaire (CIQ, described in more detail later). The mean (sd) total score on the CIQ was 16.09 (sd = 4.20) [28]. An assumed clinical relevant difference between groups of at least 10%, an alpha of 0.05 and a power (1-β) of 0.8 would necessitate 84 patients in each group. With an estimated loss to follow-up of 15%, based on earlier research in cardiac arrest survivors, 200 participants are thought to be necessary for the clinical trial [19,21,29]. This should be achievable within three years, as the area served by the participating hospitals covers approximately 1,000,000 inhabitants.

### Recruitment and randomisation

Between three and ten days after the cardiac arrest the patient and caregiver will be approached to participate in the study by their treating physician. Standard procedures concerning informed consent will be used. Both patients and caregivers sign an informed consent form if they decide to participate in the study. In case the patient is still unconscious or incapacitated at the moment that informed consent is needed, the caregiver will be asked to give provisional informed consent. When the patient regains consciousness, definitive informed consent will be sought.

Participants will be randomly allocated to either the intervention group or the control group. The randomisation will take place after the baseline measurements (2 weeks after the cardiac arrest) and will be performed centrally by the project leader using a computerised block randomisation. The randomisation scheme includes prestratification on two variables, namely hospital site and location of the cardiac arrest (in-hospital versus out-of hospital). Two series of opaque numbered envelopes will be prepared for each hospital site (one for in-hospital and one for out-of-hospital cardiac arrests), filled with a card indicating the allocated group. After registration, the envelope will be opened and the specialised nurse will be informed about the patients that are allocated to the intervention group.

### Group allocation and intervention

The intervention group will receive an early intervention service delivered by a specialised nurse in addition to usual health care available to all patients, whereas the control group will only receive usual health care. The intervention period will extend up to three months after the cardiac arrest. During this period a specialised nurse will provide one to six consultations for the patient together with his or her caregiver (if present). The number of consultations will be tailored for each individual participant and will depend on the severity of the problems and the needs and wished of the patient and their caregiver. The consultations will take place in the hospital or at the participants' home. In between the consultations the participants have the opportunity to contact the nurses by phone or email. During the intervention period the specialised nurse will screen for cognitive and emotional problems. Next to that, information will be provided by means of an information booklet and by discussing questions that the participants may have. Furthermore, emotional and practical support will be offered and self-management will be explained and promoted. A consultant in rehabilitation medicine supervises the specialised nurses. If necessary, referral to additional diagnostics or specialised rehabilitation care can take place.

There will be no restrictions on possible co-interventions. Participants assigned to the intervention group who do not want treatment will still be approached for all the follow-up measurements.

### Data collection

Baseline measurements will be administered two weeks (= T1) after the cardiac arrest, with further assessments at three (= T2) and twelve months (= T3). The measurements will be administered wherever the patient is at that time. A research assistant, who will be blinded to the group allocation, will perform the assessments.

To check for the effectiveness of blinding, the research assistants will be asked after the last measurement to indicate whether they think the participant belonged to the intervention group or the control group.

### Socio-demographical and medical variables

The following socio-demographical variables will be recorded: Age, gender, nationality, marital status, living condition, educational level, and work situation. The variables related to the resuscitation and medical situation of the patient that will be recorded are presented in Table 2.

**Table 2:** Variables related to the resuscitation and the medical condition of the patient.

Location of cardiac arrest
Cardiac arrest witnessed
Direct start CPR
Use of external automated defibrillator (AED)
Number of defibrillations
Time intervals
- collapse – start CPR
- call – response
- collapse – first defibrillation
- collapse – return of spontaneous circulation
Glasgow Coma Score at admission
Initial cardiac rhythm
Presumed aetiology cardiac arrest
Cardiac treatment during hospital admission e.g. mild therapeutic hypothermia, PTCA, CABG, ICD
Ejection fraction at discharge
Duration of coma
Duration post-traumatic amnesia
Cardiovascular history
Co-morbidity

### Outcome measures for the effect evaluation

The primary outcome measures of this trial are participation in society and quality of life of the cardiac arrest survivor. The secondary outcome measures are cognitive, emotional and cardiovascular impairment and daily functioning of the cardiac arrest survivor, as well as the strain, emotional functioning and quality of life of the caregiver. The instruments that will be used to measure these domains are presented in Table 3 and Table 4 and are described in the next sections.

**Table 3:** Outcome domains, measurement instruments and measurement moments for the cardiac arrest survivors

			Measurement moments			
**Domain**	**Measurement instrument**	**Abbr.**	**T0**	**T1**	**T2**	**T3**

Participation in society	Community Integration Questionnaire	CIQ	X		X	X
	Impact on Participation and Autonomy	IPA				X
Quality of life	Short-Form-36 (RAND 36-item Health Survey)	SF-36		X	X	X
	EuroQol 6D	EQ-6D		X	X	X
	Quality Of Life after Brain Injury	QOLIBRI			X	X
Cognitive functioning	Cognitive Failures Questionnaire	CFQ		X	X	X
	Cognitive Log	Cog-log		X	X	X
	Adult Memory and Information Processing Battery	AMIPB		X	X	X
	Paragraph Recall	PR		X	X	X
	Trail Making Test, part A + B	TMT		X	X	X
	Verbal Fluency Test	VFT		X	X	X
Emotional functioning	Hospital Anxiety and Depression Scale	HADS		X	X	X
	Impact of Event Scale	IES		X	X	X
Cardiorespiratory functioning	New York Heart Association Classification	NYHA		X	X	X
Fatigue	Fatigue Severity Scale	FSS			X	X
Basic daily activities	Barthel Index	BI		X		
Instrumental daily activities	Frenchay Activity Index	FAI	X		X	X

T0 = situation prior to cardiac arrest (asked retrospectively at T1)

T1 = 2 weeks after cardiac arrest

T2 = 3 months after cardiac arrest

T3 = 1 year after cardiac arrest

**Table 4:** Outcome domains, measurement instruments and measurement moments for the caregivers

			Measurement moments		
**Domain**	**Measurement instrument**	**Abbr.**	**T1**	**T2**	**T3**

Quality of life	Short-Form-36 (RAND 36-item Health Survey)	SF-36	X	X	X
	EuroQol 6D	EQ-6D	X	X	X
Emotional functioning	Hospital Anxiety and Depression Scale	HADS	X	X	X
	Impact of Event Scale	IES	X	X	X
Fatigue	Fatigue Severity Scale	FSS		X	X
Caregiver Strain	Caregiver Strain Index	CSI	X	X	X

T1 = 2 weeks after cardiac arrest

T2 = 3 months after cardiac arrest

T3 = 1 year after cardiac arrest

### Primary outcome measures

#### Community Integration Questionnaire

The Community Integration Questionnaire (CIQ) is a 15-item questionnaire that assesses the level of participation in society [30]. The CIQ comprises three scales, namely home integration, productive activity, and social integration.

#### Impact on Participation and Autonomy

The Impact on Participation and Autonomy (IPA) is a 39-item questionnaire that focuses on two aspects of participation, namely perceived participation and the experience of problems [31].

#### EuroQol 6D

The EuroQol 6D (EQ-6D) is a generic quality of life measure consisting of 6 items (mobility, self-care, usual activities, pain/discomfort, anxiety/depression and cognition) each with three answer possibilities [32]. In addition, participants are asked to give a general rating of their current health state on a visual analogue scale, ranging from 0 to 100.

#### Medical Outcomes Study 36-item Short Form Health Survey

The Medical Outcomes Study 36-item Short Form Health Survey (SF-36/RAND 36-item Health Survey) is a 36-item generic quality of life questionnaire that measures several domains of perceived health [33].

#### Quality Of Life after Brain Injury

The Quality Of Life after Brain Injury questionnaire (QOLIBRI) is a brain injury specific quality of life measure. The QOLIBRI consists of 49 questions that measure health related quality of life within six domains (physical condition, thinking activities, feelings and emotions, functioning in daily life, relationships and social/leisure activities, current situation and future prospects) [34].

### Secondary outcome measures

#### Cognitive Failures Questionnaire

The Cognitive Failures Questionnaire (CFQ) is a 25-item questionnaire on self-reported cognitive failures [35]. Both the patient as well as his or her caregiver will be asked to fill out this questionnaire to evaluate the cognitive status of the patient.

#### Cognitive Log

The Cognitive Log (Cog-log) is a 10-item cognitive screening instrument [36]. It measures higher neurocognitive processes including orientation, memory, concentration and executive skills.

#### Adult Memory and Information Processing Battery Task A

The Adult Memory and Information Processing Battery (AMIPB) Task A is a measure for information processing speed [37]. In this test, the subject is asked to select the second highest number in each row of five. The final score is the number of correct choices made in two minutes.

#### Paragraph Recall

The Paragraph Recall (PR) is a test for immediate and delayed verbal memory [38]. The subject is asked to recall as much as possible from a paragraph, which is read to the subject aloud. This will be performed both directly, and, without warning, again 20 minutes later. To prevent interference with the paragraph from the previous measurement, a different paragraph will be used each time.

#### Trail Making Test

The Trail Making Test (TMT) measures scanning, visuomotor tracking, divided attention and cognitive flexibility [39]. The test is divided into two parts, part A and B. In part A, the subject is asked to connect consecutively numbered circles on one work sheet as fast as possible. In part B, the subject is asked to connect consequently numbered and lettered circles. The time needed to complete part A and part B is recorded.

#### Verbal Fluency Test

During the Verbal Fluency Test (VFT), the subject is asked to name as many words from one semantic category, in this case animals, in one minute [40].

#### Hospital Anxiety and Depression Scale

The Hospital Anxiety and Depression Scale (HADS) is a commonly used questionnaire, which was designed to detect the presence of mild degrees of mood disorders in non-psychiatric hospital outpatients [41]. The HADS consists of 14 items, and has two sub-scales, namely depression and anxiety.

#### Impact of Event Scale

The Impact of Event Scale (IES) measures psychological reactions that can take place after a traumatic event. The IES has 15 items and explores the level of posttraumatic stress by asking for intrusive and avoidance symptoms [42,43].

#### New York Heart Association Classification

The New York Heart Association Classification (NYHA-classification) is a functional classification system that divides cardiac patients into four classes depending on their limitation in physical activities [44].

#### Fatigue Severity Scale

The Fatigue Severity Scale (FSS) is a 9-item questionnaire that measures experienced severity of fatigue symptoms in daily activities [45].

#### Barthel Index

The Barthel Index (BI) is a widely used instrument that measures basic (personal) activities of daily living (ADL). The instrument consists of 10 items and measures to what extent a person can perform basic ADL activities independently [46].

#### Frenchay Activities Index

The Frenchay Activities Index (FAI) is an accepted measure for instrumental ADL that reports on fifteen daily life activities [47]. Compared to the Barthel Index, the measured activities are of a higher level, for example the preparation of meals, doing groceries, making trips and performing paid labour.

#### Caregiver Strain Index

The Caregiver Strain Index (CSI) is a 13-item, dichotomous scale that has to be filled out by a partner, caregiver or significant other [48]. The CSI measures strain related to the provision of care.

### Parameters for process evaluation

The intervention will be evaluated based on information from the specialised nurses and the participants. First, the intervention process will be evaluated based on information provided by the specialised nurse. He/she will record the following items: the actual number of consultations (face-to-face, by telephone and by email), the direct time related to each consultation, the indirect time related to each consultation (administration, travel time), the provision of information per consultation, the use of the screening tools, the kind of problems presented, number of referrals, completion of the intervention according to protocol and, in case of non-compliance, reason for non-compliance. Second, after completion of the intervention the participant and his/her caregiver will be asked to fill out a questionnaire to evaluate the intervention.

### Determination of costs

Data concerning costs will be gathered prospectively using monthly cost-diaries [49]. All the participants receive cost-diaries in which they are asked to note all their health care utilisation during that month. To prevent non-compliance, the research assistant will give a phone call at the end of each month and will record the health care utilisation of that month. For the calculation of the total costs, three categories can be distinguished, namely direct health care costs, direct non-health care costs and indirect costs. For the determination of the direct health care costs the following variables will be recorded: duration admission in health care facility, visits physicians and other health care providers and amount of prescribed medication. Direct non-health care costs that will be measured, are the costs of informal health care, over-the-counter medication, costs of health activities, hours of paid or unpaid household help and special aids. The indirect costs comprise loss of paid and unpaid work productivity of the patient.

### Statistical analysis

#### Effect evaluation

Descriptive techniques will be used to present data concerning the participants, number of dropouts, losses-to-follow up and the scores on the outcome measures.

To determine whether the patients participating in this trial are representative for the population of cardiac arrest survivors, the baseline characteristics between compliant and non-compliant participants, as well as dropouts and losses-to-follow up will be compared.

Before examining the effectiveness of the intervention, the comparability of the two groups will be checked. Baseline characteristics will be compared using independent sample T-test (normal distribution) or Mann Whitney U-test (non-normal distribution), in case of a continuous variable. In case of a dichotomous variable a chi-square test will be used.

The data from the evaluation study will be analysed according to the intention-to-treatment principle. If either T1 or T2 data are missing the 'last-observation-carried-forward' principle will be used.

Multiple regression analysis, adjusted for possible differences in baseline characteristics or baseline scores, will be applied to examine differences between the intervention and the control group on the primary outcome measures at T3 (one year after the cardiac arrest). If there is a difference between the groups, the effect size will be calculated. Subgroup analysis will be performed for potential effect modifiers.

To assess whether protocol deviations or care provided outside the intervention have caused biases, the results of the intention-to-treatment analysis will be compared to the on-treatment analysis.

#### Process evaluation

The outcomes of the process evaluation will be reported using descriptive techniques.

#### Economic evaluation

To determine the cost-effectiveness of the intervention a statistical analysis of costs will be performed. The total costs are calculated by adding up direct health care costs, direct non-health care costs and indirect costs. Health care costs are estimated using the Dutch guideline for cost analysis in health care research [50]. The Friction Cost Approach will be used to evaluate production losses, necessary to determine indirect costs [50]. Discounting of costs will take place if significant changes in health care costs take place during the study period. As the distribution of costs might be skewed, differences in costs between the intervention and care-as-usual will be calculated by means of bootstrapping, a method appropriate for any distribution of data.

A multi-way sensitivity analysis will be performed to gain insight into the generalisability of the economic evaluation.

A cost-utility analysis will relate the difference in between the intervention and care-as-usual to changes in utility. This will result in costs per quality-adjusted-life-years (QALY's).

## Discussion

The main goal of the ALASCA trial is to evaluate a new early intervention service for cardiac arrest survivors and their caregivers. This is a relevant topic because evidence on effective rehabilitation programmes for cardiac arrest survivors is urgently needed. During the design of this study many choices and selections were made, and four of the more important ones will be discussed.

First, the intervention was designed especially for this study. There are very few studies describing programmes for people with hypoxic brain injury due to a cardiac arrest. Consequently, we chose to model the intervention upon evidence of effective interventions for people who had traumatic brain injury, as there are many similarities between hypoxic and traumatic brain injuries [27]. This intervention designed for this study is a combination of a psychosocial intervention and a process intervention. The psychosocial aspects of the intervention are directed at providing informational, emotional and practical support and simulating self-management. The intervention is also process intervention because it is directed at providing the care needed fast and efficiently to each individual patient.

Second, the selection of our primary outcome measures, participation in society and quality of life, may differ from common primary outcome measures in cardiology research. However, this is a common approach in rehabilitation medicine, where maximisation of participation in society is one of the main goals [51]. We expect that through the care provided in the new intervention, patients will be able to reach a higher level of participation, as well as a higher quality of life.

Third, it may need some clarification why three quality of life measures will be administered. The EQ-6D and the SF-36 were chosen because they measure generic quality of life and can be used in the cost-effectiveness analyses. Next to that, these questionnaires have been used in many other patient groups, which makes it possible to compare the quality of life of cardiac arrest survivors to that of other patient groups. The third quality of life measure is the QOLIBRI, which is a brain injury specific questionnaire. This questionnaire was included because it may have a higher sensitivity to change in case of hypoxic brain injury than both general quality of life measures.

Fourth, we explicitly chose to include the strain for and quality of life of the caregiver in the secondary outcome measures. Our impression is that being the partner of a cardiac arrest survivor can imply a heavy burden. These caregivers are at risk for developing stress and emotional problems and this definitely needs more attention. Therefore, the intervention is directed at both the patient and their caregiver, who is expected to benefit from the early intervention service as well.

To conclude, this paper describes the design of a randomised, controlled clinical trial that will investigate the effectiveness of a new early intervention service for cardiac arrest survivors and their caregivers. The inclusion of the participants started in April 2007 and will continue until April 2010. The results of this study will provide evidence on the effectiveness of this early intervention service, as well as the cost-effectiveness and its feasibility. This will give insight into the question whether it would be useful to implement this early intervention service in the Dutch health care system.

## Competing interests

The author(s) declare that they have no competing interests.

## Authors' contributions

VRMP is the main researcher and is responsible for writing the protocol. JAV and WGMB originated the idea for the study. JAV is project-leader of the study. JAV and CMvH are the supervisors of VRMP. DTW is the promotor of VRMP.

JAV, CMvH, WGMB, APMG, SCAMB, MCFTMdK and DTW participated in the design of the study and research protocol. All authors read and corrected draft versions of the manuscript and approved the final manuscript.

## Pre-publication history

The pre-publication history for this paper can be accessed here:


